# Evidence for a Role for Interleukin-17, Th17 Cells and Iron Homeostasis in Protective Immunity against Tuberculosis in Cynomolgus Macaques

**DOI:** 10.1371/journal.pone.0088149

**Published:** 2014-02-04

**Authors:** Alice S. Wareham, Julia A. Tree, Philip D. Marsh, Philip D. Butcher, Mike Dennis, Sally A. Sharpe

**Affiliations:** 1 Public Health England, Microbiology Services, Porton Down, Salisbury, United Kingdom; 2 Department of Medical Microbiology, St. George’s Hospital Medical School, London, United Kingdom; Colorado State University, United States of America

## Abstract

Tuberculosis (TB) remains a major global public health problem. The only vaccine, BCG, gives variable protection, especially in adults, so several new vaccines are in clinical trials. There are no correlates of protective immunity to TB; therefore vaccines progress through lengthy and expensive pre-clinical assessments and human trials. Correlates of protection could act as early end-points during clinical trials, accelerating vaccine development and reducing costs. A genome-wide microarray was utilised to identify potential correlates of protection and biomarkers of disease induced post-BCG vaccination and post-*Mycobacterium tuberculosis* challenge in PPD-stimulated peripheral blood mononuclear cells from cynomolgus macaques where the outcome of infection was known. Gene expression post BCG-vaccination and post challenge was compared with gene expression when the animals were naïve. Differentially expressed genes were identified using a moderated T test with Benjamini Hochberg multiple testing correction. After BCG vaccination and six weeks post-*M. tuberculosis* challenge, up-regulation of genes related to a Th1 and Th17 response was observed in disease controllers. At post-mortem, RT-PCR revealed an up-regulation of iron regulatory genes in animals that developed TB and down-regulation of these genes in disease controllers, indicating the ability to successfully withhold iron may be important in the control of TB disease. The induction of a balanced Th1 and Th17 response, together with expression of effector cytokines, such as IFNG, IL2, IL17, IL21 and IL22, could be used as correlates of a protective host response.

## Introduction

Tuberculosis (TB) is still a major global public health issue [1]. The exact mechanisms of protection induced by the only vaccine currently licensed, BCG, have not been fully elucidated and the development of new interventions has been hindered by the lack of a definitive correlate of protection. Therefore, potential TB vaccine candidates have to be tested empirically for “protection” in a range of animal models, from which the more promising vaccines are selected for progression into lengthy and expensive clinical trials, often involving numerous at-risk individuals in endemic countries. The identification of correlates of protection for TB would prove beneficial in the development of a new vaccine in several ways. A validated correlate of protection could be used in screening new vaccine candidates to ascertain that they generate an appropriate immune response and also be used as a surrogate end-point during clinical trials, substituting for a clinical end-point. This would accelerate clinical trials and vaccine evaluation in pre-clinical studies, which would not only reduce costs but also allow the assessment of a greater number of vaccine candidates in a set period of time. Much time, money and research has gone into trying to clarify the interactions that occur between pathogen and host, and through this analysis, it has become evident that the immune response to *Mycobacterium tuberculosis* is a complex and multifaceted process. Studies have shown the importance of the Th1 immune response and cytokines, such as interferon gamma (IFNG), interleukin (IL)-12 and tumor necrosis factor (TNF) in the control of TB disease [2]–[4]. IFNG, in particular, is essential for protective immunity to tuberculosis, and individuals who carried a genetic deficiency in the receptor for IFNG were more likely to succumb to mycobacterial infection [2], [5]. IFNG was once proposed as a potential correlate of protection, but it is now apparent that IFNG alone is not a reliable correlate as its production before and during a mycobacterial infection is not predictive of protection against disease [6]–[8] and it is likely that other factors, possibly ones that are regulated by the cytokine, may be involved [9]. Other aspects of the immune response proposed to play a role in protection against TB include CD8+ T cells, Th17 cells, γδ T cells, CD1-restricted invariant Natural Killer T (iNKT) cells and mucosal-associated invariant T (MAIT) cells [10]–[14]. Several methods have been employed to assess responses induced by vaccination and challenge. ELISAs and Luminex® technology are used to detect cytokines including IFNG, the ELISPOT is used to determine the frequency of antigen-specific T cells, and flow cytometry can be used to assess cell surface markers and intracellular cytokines. However, resources are often limited so not all of these technologies can be applied. Choices need to be made; therefore bias is introduced into the analysis. A whole-genome approach may be a more realistic method by which to identify correlates of protection as aspects of all areas of the immune system can be examined together, along with other facets of the response which might currently be considered unrelated, or unimportant. Transcriptomics provides a way to do this and has been used to identify genes associated with BCG-vaccination success in mice [15].

As our closest relatives phylogenetically, non-human primates (NHPs) have been utilised to model tuberculosis infection because they are naturally susceptible to *M. tuberculosis*, share clinical and pathological changes caused by TB in humans, and are also protected to varying degrees by BCG vaccination. The NHP model has been used to evaluate vaccine-induced immunity to tuberculosis [16]–[19]. Data derived from studies in NHPs are believed to hold strong predictive validity and support the development of the more promising vaccine candidates. An advantage of the NHP model is that sequential samples can be taken, providing the opportunity for longitudinal analyses, which is not feasible in smaller animal models. In addition, other factors, such as infective dose and time from infection can be controlled, which is not possible in human studies. Therefore, the NHP model provides a definable experimental approach to identifying biomarkers.

In this study, microarray technology has enabled a genome-wide examination of gene expression in response to BCG-vaccination and TB infection by utilising an archive of peripheral blood mononuclear cells (PBMCs) isolated and cryopreserved during a vaccine efficacy study in NHPs where the outcome of infection was known. Due to the number of animals involved in the original study, the aim of this analysis was to generate biology-driven hypotheses from the molecular data, and fresh insights with which to inform future studies, rather than identifying a distinctive biosignature of protection or disease as has been carried out in other studies [45]. Deciphering mechanisms of host-mediated immune responses to this highly adapted intracellular pathogen in the cynomolgus macaque model of TB, by identifying responses indicative of protection or disease progression, could potentially provide an important step forward for developing and evaluating new interventions.

## Methods

### Ethics Statement

Animals were housed according to the Home Office (UK) Code of Practice for the Housing and Care of Animals Used in Scientific Procedures (1989) and the National Committee for Refinement, Reduction and Replacement (NC3Rs) Guidelines on Primate Accommodation, Care and Use, August 2006, for the care and maintenance of primates. All protocols involving animals were approved by the Ethical Review Committee of the Public Health England, Porton, UK. Following local ethical review, project licence 30/2565 was subsequently granted by the UK Home Office.

### Experimental Animals

Twelve male captive-bred cynomolgus macaques of Chinese origin were used for this study. CITES permit and Home Office approval were obtained prior to importation. Animals were housed in social same-sex groups of 4 to 6 in cages that met with the UK Home Office Code of Practice and the European Standard ETS123. Animals were given ad libitum access to sterile bottled water and primate expanded mazuri pellets (SQC). This was supplemented by forage mix, fresh fruit and vegetables at regular intervals. Environmental enrichment was provided by a varied programme that included toys, puzzle feeders and DVDs for visual stimulation. All animals underwent full veterinary health inspection on arrival and there were subsequent daily checks on clinical signs and behaviour. At 2 week intervals each animal was examined under sedation, weighed and a blood sample was taken for immunological and health screening.

All animals were 4 years old at the time of challenge, weighing between 2.8–4.4 Kg, and naïve in terms of prior exposure to mycobacterial antigens (*M. tuberculosis* infection or environmental mycobacteria) as demonstrated by the tuberculin test whilst in their original breeding colony and by the IFNG based Primagam test kit (Biocor, CSL, USA) just prior to study start. Monkeys were sedated by intramuscular (i.m.) injection with ketamine hydrochloride (10 mg/kg) (Ketaset, Fort Dodge Animal Health Ltd, Southampton, UK) for all procedures requiring removal from their cages. None of the animals had been used previously for experimental procedures. The time of necropsy if prior to the end of the planned study period was determined by experienced primatology staff based on a combination of the following adverse indicators: depressed or withdrawn behaviour, dyspnoea, loss of 20% of peak post-challenge weight, erythrocyte sedimentary rate (ESR) levels elevated above normal, haemoglobin level below normal limits, increased temperature and severely abnormal chest X-ray. At termination animals were anaesthetised with ketamine at 0.2 ml/Kg and then killed with an overdose of pentobarbitone sodium.

### BCG-Vaccination

**Figure 1:**
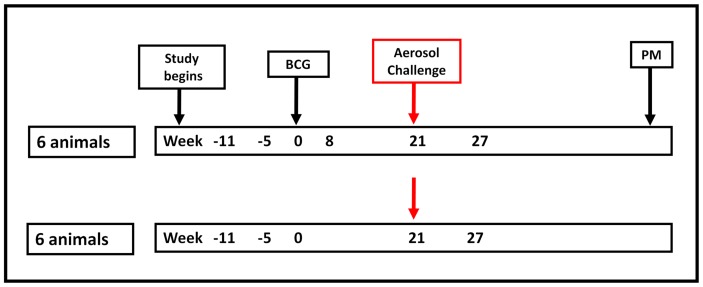
Vaccination and challenge schedule. Twelve Chinese cynomolgus macaques were involved in the study. Six animals received BCG-vaccination and six animals remained unvaccinated prior to aerosol challenge with *M. tuberculosis*. All animals were followed for a maximum of 28 weeks post-infection. Time-points at which gene expression analysis was carried out are shown in bold. PM = post-mortem.

A plan of the vaccination strategy and timing of the aerosol challenge is shown in Figure 1. Six of the twelve Chinese cynomolgus macaques were immunised intradermally in the upper left arm with 100 µl BCG, Danish strain 1331 (SSI, Copenhagen, Denmark). The BCG vaccine was prepared and administered according to manufacturer’s instructions for preparation of vaccine for administration to human adults, by addition of 1 ml Sauntons diluent to a vial of vaccine, to give a suspension of BCG at an estimated concentration of 2×10^6^ to 8×10^6^ CFU/ml.

### *M. tuberculosis* Challenge

Twenty-one weeks after immunisation with BCG, the six BCG-immunised animals together with six unvaccinated animals were challenged by the aerosol route with *M. tuberculosis* Erdman strain K 01, kindly provided by Dr Amy Yang (CBER/FDA now BEI Resources), (Figure 1) as previously described [20]. The process to deliver a known number of viable *M. tuberculosis* bacilli in a target volume of inspired aerosol was performed. Challenge was performed on sedated animals placed within a “head-out” plethysmography chamber (Buxco, Wilmington, NC) to enable the aerosol to be delivered simultaneously with the measurement of the respiration rate. Animals received an aerosol dose of 75 CFU and were monitored for a maximum of 28 weeks post infection (p.i.).

### Isolation and Stimulation of PBMCs

Peripheral blood mononuclear cells (PBMC) were isolated from heparin-anticoagulated blood by Ficoll-Hypaque Plus (GE Healthcare, Buckinghamshire, UK) density gradient separation using standard procedures. PBMCs were isolated from two time-points when the animals were naïve, at 8 weeks post-vaccination in animals that received BCG, and at 6 weeks post-*M. tuberculosis* challenge in all animals and cryopreserved in 10% DMSO/90% foetal bovine serum, on day of isolation. Time-points were selected based on immunological responses observed in ELISPOT, IFNG ELISA and whole blood intracellular staining gathered during the vaccine efficacy study (data not shown) where peak responses to BCG vaccination were observed around 8 weeks post-vaccination. Six weeks post-challenge was selected as it allowed time for an adaptive immune response to be mounted but before inflammation or infection could overwhelm the gene expression signal. Later, PBMCs were thawed, washed and resuspended in medium consisting of RPMI 1640 supplemented with L-glutamine (2 mM), penicillin (50 U/ml)/streptomycin (50 µg/ml) and 10% heat-inactivated foetal bovine serum with 50 U/ml Benzonase (Scientific Laboratory Supplies Ltd, Nottingham, UK ), pre-warmed to 37°C and “rested” for 2 hours at 37°C in 5% CO_2_. After resting, cells were washed and cell concentrations adjusted to 2×10^6^ cells/ml in medium. PBMCs were stimulated with 30 µg/ml purified protein derivative (PPD) (SSI, Copenhagen, Denmark) for 16 hours at 37°C in the presence of 5% CO_2_.

### RNA Isolation and Purification

RNA was isolated from PBMC samples using Trizol reagent (Invitrogen, Paisley, UK) as described in the manufacturer’s protocol, and RNA purified using RNeasy spin columns (Qiagen, Crawley, UK) including an on-column DNA digest step (Qiagen, Crawley, UK). RNA was quantified using a NanoDrop ND1000 UV-vis spectrophotometer (NanoDrop Technologies, Cambridge, UK), and the quality assessed using the Agilent 2100 Bioanalyzer (Agilent Technologies, Stockport, UK). All RNA samples used for microarray and real-time PCR (RT-PCR) analysis had an RNA Integrity Number (RIN) of at least 7.0.

### Microarray Procedure

50 ng of RNA extracted from PPD-stimulated PBMCs was used for microarray analysis. RNA was amplified and labelled using the Agilent Low Input Quick Amplification Labelling kit (Agilent Technologies, Stockport, UK) with RNA spike-ins (Agilent Technologies, Stockport, UK), as per kit instructions. cRNA was purified using the Qiagen RNeasy mini columns (Qiagen, Crawley, UK) and the yields and dye-incorporation rate were measured using a NanoDrop ND1000 UV-vis spectrophotometer. The hybridization procedure was performed according to the Agilent 60-mer oligo microarray processing protocol using the Agilent Gene Expression Hybridization Kit. Briefly, 1.65 µg Cy3-labelled fragmented cRNA in hybridization buffer was hybridized overnight (17 hours, 65°C, 10 rpm) to Agilent Rhesus Macaque Gene Expression Microarrays 4×44 K (Array ID-15421) using Agilent’s recommended hybridization chamber and oven. Slides were washed and scanned using the Agilent’s High-Resolution C Scanner at a resolution of 5 µm. Data were extracted using Agilent Feature Extraction software v10.7 using the default settings for 1-colour gene expression analysis (protocol GE1_107_Sep09), which determines feature intensities, subtracts background signal, rejects outliers and calculates statistical confidences. Complete microarray data are available at the Gene Expression Omnibus (GEO) database under the accession number GSE42273.

### Data Analysis

Initial data analysis was carried out using GeneSpring GX12 (Agilent Technologies, Stockport, UK). Data underwent quantile normalisation and the expression profiles post-vaccination and post-challenge were baseline transformed to expression at the naïve time-points for each animal. Samples were grouped according to immune status (naïve, post-vaccination, post-challenge), vaccination status (vaccinated, unvaccinated) and whether the animal was able to control infection for the duration of the study or not (controllers, progressors). Gene expression post-vaccination and post-challenge was compared to gene expression at the naïve time-points for each group in pair-wise comparisons. Genes were filtered according to expression and fold-change so that only genes which were detected in at least 50% samples in 1 of the 2 conditions i.e. naïve or post-vaccination/post-challenge and had a fold change of at least 1.75 at post-vaccination and 2.00 at post-challenge, were used for further analysis. A moderated T-test with Benjamini-Hochberg false discovery rate (FDR) correction for multiple testing was performed to identify differentially expressed genes with a corrected p-value threshold of 0.05 for the post-vaccination time-point and 0.01 for the post-challenge time-point. The more stringent limits were used for the post-challenge time-point as a greater number of genes were found to be differentially expressed so analysis was focused on the most significant genes exhibiting greatest fold changes in expression (fold change >2, corrected p-value <0.01).

### Real-time PCR

A subset of genes was selected for further investigation and for validation of expression by RT-PCR. The High Capacity RNA-to-cDNA kit (Applied Biosystems, Warrington, UK) was used to reverse transcribe RNA extracted from PBMCs as per kit instructions and cDNA was stored at −20°C until use. Pre-designed and pre-optimized Taqman® gene expression assays and TaqMan® Gene Expression Master Mix were used with the Applied Biosystems 7900HT Real-Time PCR system. All assays were designed to span exon boundaries. Assay ID were Rh02847367 (B2M), Rh02842097_m1 (CXCL9), Rh02789784_m1 (FTH1), Rh02621747_m1 (HMOX1), Rh02788577_m1 (IFNG), Rh02789780_m1 (IL2), Rh02789322_m1 (IL6), Rh02621748_m1 (IL12B), Rh02621750_m1 (IL17), Rh02879198_m1 (IL21), Rh02877345_m1 (IL22), Rh02872166_m1 (IL23A), Rh02796598_m1 (SLC11A1), Rh02829242_m1 (SLC11A2), Rh02621758_m1 (TFRC), Rh02789784_m1 (TNF). Reactions were run in triplicate and the fold increase was calculated using the relative quantification method, with B2M used as the endogenous control and the PPD-stimulated naïve samples for each animal used as the calibrator sample. All RT-PCR data were analysed using the ABI SDS 2.4 and RQ manager 1.2.1 software. Significant differences between expression profiles were determined by Student T tests carried out on the log_2_-transformed RT-PCR data; a p-value of <0.05 was taken as statistically significant using SigmaPlot 11.0.

### Role of Iron Homeostasis in TB Disease

Microarray data highlighted a potential role of iron homeostasis in the control of TB disease. This was investigated further by analysing the expression of five genes involved in the control of cellular iron in unstimulated PBMCs isolated from the six unvaccinated cynomolgus macaques at post-mortem, using RT-PCR. At the point of necropsy, the animals had either succumbed to infection and TB disease had progressed to reach humane end-point criteria, or had managed to control the infection showing little or no overt symptoms of disease.

## Results

### Outcome of Challenge and Grouping of Animals

All twelve animals involved in the vaccine efficacy study were challenged by the aerosol route with *M. tuberculosis*. The six animals that received BCG prior to challenge all controlled the infection for the duration of the study (28 weeks post-challenge). This group is referred to as “vaccinated controllers”. Three of the unvaccinated animals were also able to control infection for the duration of the study and are referred to as “unvaccinated controllers”. The remaining three unvaccinated animals developed active TB disease and reached humane end-point criteria before the end of the study and are referred to as “unvaccinated progressors”.

### Differentially Expressed Genes Identified Following BCG-Vaccination

Using a fold change cut off of at least 1.75 and a corrected p-value cut off of 0.05, a list of 13 genes were found to be significantly differentially expressed 8 weeks after BCG-vaccination in the 6 animals that received BCG (Table 1). The gene exhibiting the greatest increase in expression was the cytokine *CXCL9*. Also within the list were the genes expressing the closely related chemokines *CXCL10* and *CXCL11* and the Th1-associated cytokines, *IL2* and *IFNG*. In addition, the pro-inflammatory cytokine *IL17* was significantly up-regulated with a fold change of 2 from the naïve time-point.

**Table 1:** Differentially expressed genes in PPD-stimulated PBMCs isolated from cynomolgus vaccinated controllers 8 weeks post-BCG vaccination.

Agilent Probe ID	Gene Symbol	Gene Name	Fold change	Correctedp-value
A_01_P019234	*CXCL9*	chemokine (C-X-C motif) ligand 9	10.451	1.2 E-04
A_01_P005324	*CXCL11*	chemokine (C-X-C motif) ligand 11	3.939	2.6 E-04
A_01_P017394	*IL2*	interleukin 2	3.647	4.1 E-04
A_01_P016296	*CXCL10*	chemokine (C-X-C motif) ligand 10	2.402	3.2 E-03
A_01_P011873	*IL17*	interleukin 17A	2.153	1.7 E-02
A_01_P013686	*SERPING1*	serpin peptidase inhibitor, clade G 1	2.040	9.6 E-03
A_01_P000478	*IFNG*	interferon-gamma	1.988	6.0 E-03
A_01_P015977	*APOL6*	apolipoprotein L6-like	1.973	1.1 E-02
A_01_P012338	*CCL20*	chemokine (C-C motif) ligand 20	1.922	9.6 E-03
A_01_P013340	*LOC100430310*	hypothetical protein	1.873	6.0 E-03
A_01_P019222	*KLF16*	insulinoma-associated protein 1-like	1.787	4.1 E-02
A_01_P002902	*NLGN4X*	neuroligin 4	1.751	6.0 E-03
A_01_P009015	*CLEC1A*	C-type lectin domain family 1, member A	–1.800.8	6.0 E-03

Fold change indicates the change in gene expression in PPD-stimulated PBMCs isolated at 8 weeks post-BCG vaccination compared with PPD-stimulated PBMCs isolated pre-vaccination when the animals were naïve (n = 6).

### Differentially Expressed Genes Identified Following *M. tuberculosis* Challenge

The cynomolgus macaques were defined as either vaccinated controllers, unvaccinated controllers or unvaccinated progressors depending on their vaccination status at the time of challenge and whether they controlled disease for the duration of the study (28 weeks after aerosol challenge) or whether TB disease progressed sufficiently to meet humane end-point criteria.

Transcriptomic analysis of PPD-stimulated PBMCs isolated six weeks post-infection from the six vaccinated controllers revealed 705 transcripts representing 662 genes, which were significantly differentially expressed from naïve time-points using a corrected p-value of <0.01. Of these transcripts, 341 were significantly up-regulated and 364 were significantly down-regulated (Table S1).

Three cynomolgus macaques that were not vaccinated prior to challenge with *M. tuberculosis* were able to control the infection for the duration of the study. For this experimental group, 2706 transcripts, representing 2559 genes, were differentially expressed in comparison to the naïve time-point; 1233 were up-regulated and 1473 were down-regulated (Table S2).

Three cynomolgus macaques that did not receive a BCG vaccination before challenge with *M. tuberculosis* developed TB disease that met humane end-point criteria before the end of the study. In this group of animals, 1958 transcripts, representing 1828 genes, were differentially expressed in PPD-stimulated PBMCs isolated at six weeks post-challenge. Of these transcripts, 963 were up-regulated and 995 were down-regulated from the naïve time-points (Table S3).

**Figure 2:**
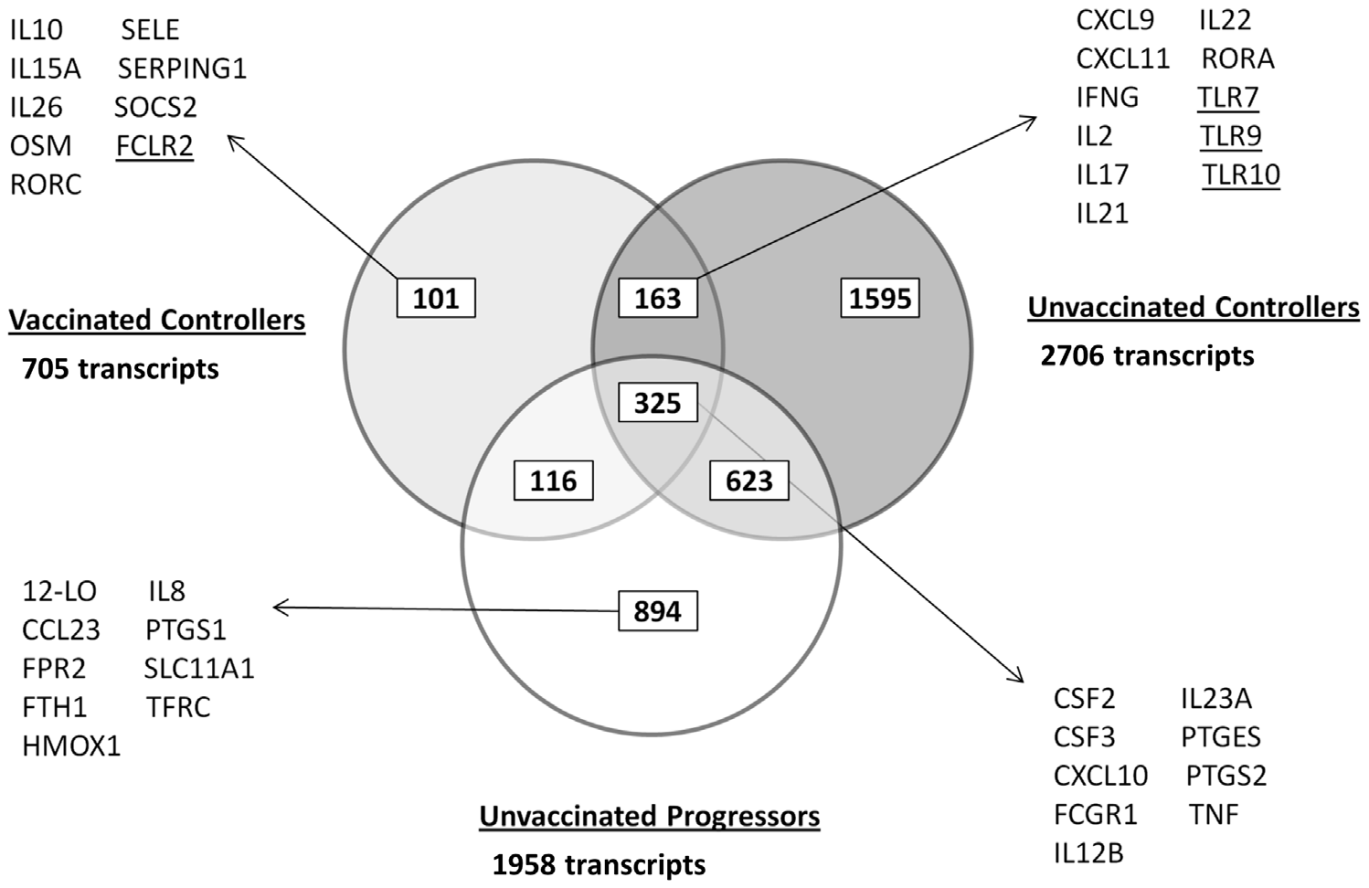
Comparison of differentially expressed gene lists across experimental groups. Microarray analysis was used to identify differentially expressed genes in PPD-stimulated PBMCs isolated at 6 weeks post-challenge with *M. tuberculosis* from vaccinated controllers (n = 6), unvaccinated controllers (n = 3) and unvaccinated progressors (n = 3) compared with expression when animals were naïve. Key genes of interest in each list are indicated. Underlined genes were down-regulated in expression from naïve time-points.

The gene lists generated from pair-wise comparisons of expression post-challenge were compared across experimental groups to determine which genes were shared and which were expressed solely by either the controllers or progressors in order to identify those which potentially correlated with protection or were biomarkers of disease (Figure 2).

### Differentially Expressed Genes Common to All Experimental Groups

Comparing the gene lists from each of the experimental groups revealed a common list of 325 genes which were differentially expressed in all groups six weeks after challenge (Figure 2); 156 genes were up-regulated and 169 down-regulated from the naïve time-point. Up-regulated genes included the chemokines and cytokines *CSF2, CSF3, CXCL10, IL12B, IL1F9, IL23A, TNF* and *TNFAIP6* and cell surface proteins macrophage scavenger receptor (MSR)-1, *CD274* and intercellular adhesion molecule (ICAM)-1. Also up-regulated across all groups were the genes expressing the enzymes prostaglandin E synthase (*PTGES*) and prostaglandin-endoperoxide synthase 2 (*PTGS2*), which are involved in the biosynthesis of prostaglandin E2 (PGE_2_) and showed the greatest increase in the animals that developed TB disease.

Of the 156 genes up-regulated across all groups, the unvaccinated progressors had 25 genes with a fold change greater than 10 compared to just 2 and 3 genes with a fold change greater than 10 for the vaccinated and unvaccinated controllers, respectively. The greatest difference was seen for the high affinity immunoglobulin gamma Fc receptor I (*FCGR1*), typically expressed on monocytes, which was increased 29 fold in the disease progressors but just 3–4 fold in the vaccinated and unvaccinated disease controllers.

### Differentially Expressed Genes in Vaccinated Cynomolgus Macaques able to Control TB Infection Only

101 transcripts were found to be differentially expressed in the cynomolgus macaques that received BCG vaccination and were able to control infection following *M. tuberculosis* challenge, which were not differentially expressed in any of the other experimental groups (Figure 2); 52 were up-regulated from the expression levels obtained when the animals were naïve and 49 were down-regulated. The gene exhibiting the greatest fold increase in expression was oncostatin M (*OSM*), which codes for a cytokine belonging to the same family of IL6, which exhibited a 6.5 fold increase from the naïve time-point. Also amongst the up-regulated genes were the Th17 lineage-specific transcription factor, RAR-related orphan receptor (ROR)-C, serpin peptidase inhibitor, clade G (C1 inhibitor) member 1 (*SERPING1*), an inhibitor of the complement cascade, selectin E (*SELE*) which mediates the adhesion of cells to the vascular lining and accumulation of leukocytes at sites of inflammation, the cytokines *IL10* and *IL26* and cytokine receptor *IL15RA*, and suppressor of cytokine signalling (SOCS) - 2. Among the down regulated genes was the Fc receptor-like 2 (*FURL*), which plays a negative immunomodulatory function in the regulation of memory B cells.

### Differentially Expressed Genes in Both Cynomolgus Vaccinated and Unvaccinated Controllers of Disease Only

163 genes were differentially expressed in animals that were able to control infection, regardless of their vaccination status (Figure 2); 46 were up-regulated from the naïve time-point and 117 down-regulated. Amongst the most up-regulated genes were the chemokines *CXCL9* and *CXCL11*, which were also amongst the most up-regulated genes after vaccination in the BCG-immunised animals. Similarly, *IL2* and *IFNG* were significantly up-regulated in both vaccinated and unvaccinated controllers post-challenge, as was *IL17*. In addition, a number of other Th17-related immune genes were differentially expressed in animals able to control TB infection including the effector cytokines, *IL21* and *IL22,* and the transcription factor, *RORA*. Amongst the down-regulated genes were phospholipase C gamma 2 (*PLCG2*) involved in T cell receptor signal transduction and the toll-like receptors (*TLR)*-7, *TLR9* and *TLR10* which exhibited fold changes of -3, -2 and -3, respectively, from the naïve time-point.

### Differentially Expressed Genes in Cynomolgus Unvaccinated Disease Progressors Only

Of the 1958 genes which were differentially expressed in the cynomolgus macaques that were unvaccinated and succumbed to disease following challenge with *M. tuberculosis,* 894 were differentially expressed by this group of animals and not in either group of animals that were able to control infection (Figure 2); 548 were up-regulated from the naïve time-point and 346 were down-regulated.

Among the up-regulated genes were *PTGS1*, *CCL23, CD14, TLR2, TLR4* and *TLR8*, the interferon associated genes *IFNB1, IFI44L* and *IFIT2* and also *12-LO* and *FPR2* which are involved in lipoxin signalling. In addition, within this gene list were a number of genes associated with iron homeostasis including ferritin heavy chain (*FTH1*), *SLC11A1*, transferrin receptor (*TFRC*), heme oxygenase 1 (*HMOX1*) and 5-aminolevulinate synthase, erythroid-specific, mitochondrial (*ALAS2*) which all exhibited up-regulation from the naïve time-point. Down-regulated genes include *TLR3, IFNA13* and *IFNA14*.

### Iron Regulation in TB Disease

Microarray analysis revealed an up-regulation of a number of iron-regulatory genes in PPD-stimulated PBMCs isolated six weeks post-challenge from animals that were unable to control TB infection, which were not significantly differentially expressed in animals that controlled disease. To further investigate the role of iron homeostasis in tuberculosis disease, RT-PCR was used to determine the expression of five genes involved in iron homeostasis in unstimulated PBMCs isolated at post-mortem in the six unvaccinated cynomolgus macaques at which point three animals had succumbed to infection and three had successfully managed to control infection for the duration of the study (28 weeks p.i).

**Figure 3:**
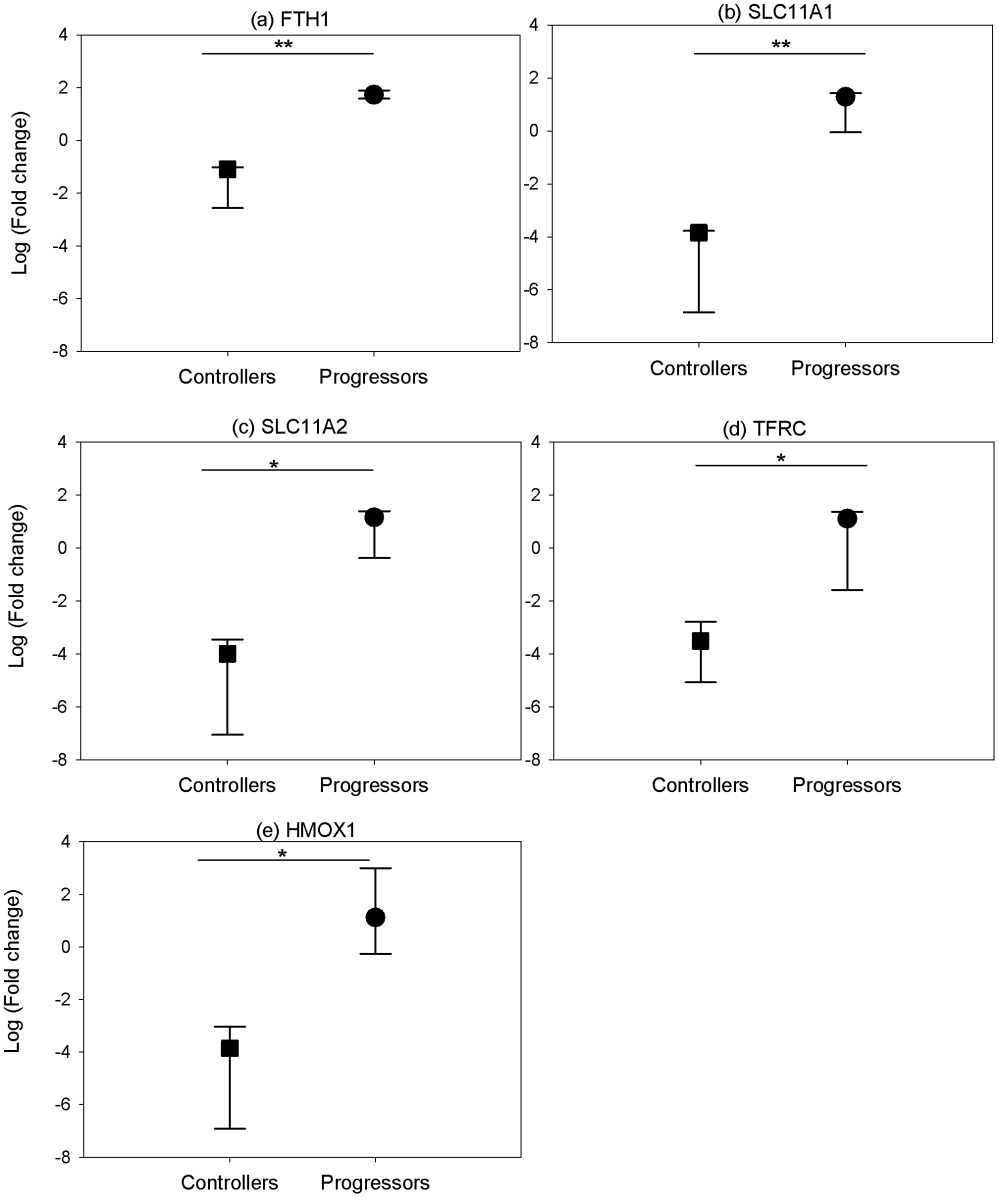
Expression of iron regulatory genes in PBMCs isolated from unvaccinated controllers and progressors at post-mortem. Gene expression of a) ferritin heavy chain b) solute carrier family 11 member A1, and c) solute carrier family 11 member A2, d) transferrin receptor 1 and e) heme oxygenase 1 in unstimulated PBMCs isolated at post-mortem. The fold change from naïve baseline levels was determined using RT-PCR. To determine statistically significant differences of relative gene expression, a two-sample t-test was performed where * and ** represent P-values of <0.05 and <0.01, respectively. The data were from 3 animals. The symbol represents the median and the error bars represent the range.

RT-PCR analysis revealed up-regulation of all of the iron regulatory genes (*FTH1, SLC11A1, SLC11A2, TFRC* and *HMOX1)* in disease progressors and a down-regulation of the same genes in disease controllers in PBMCs isolated at post-mortem, with statistically significant differences between the two groups for all 5 genes (Figure 3).

### RT-PCR Validation of Microarray Data

**Figure 4:**
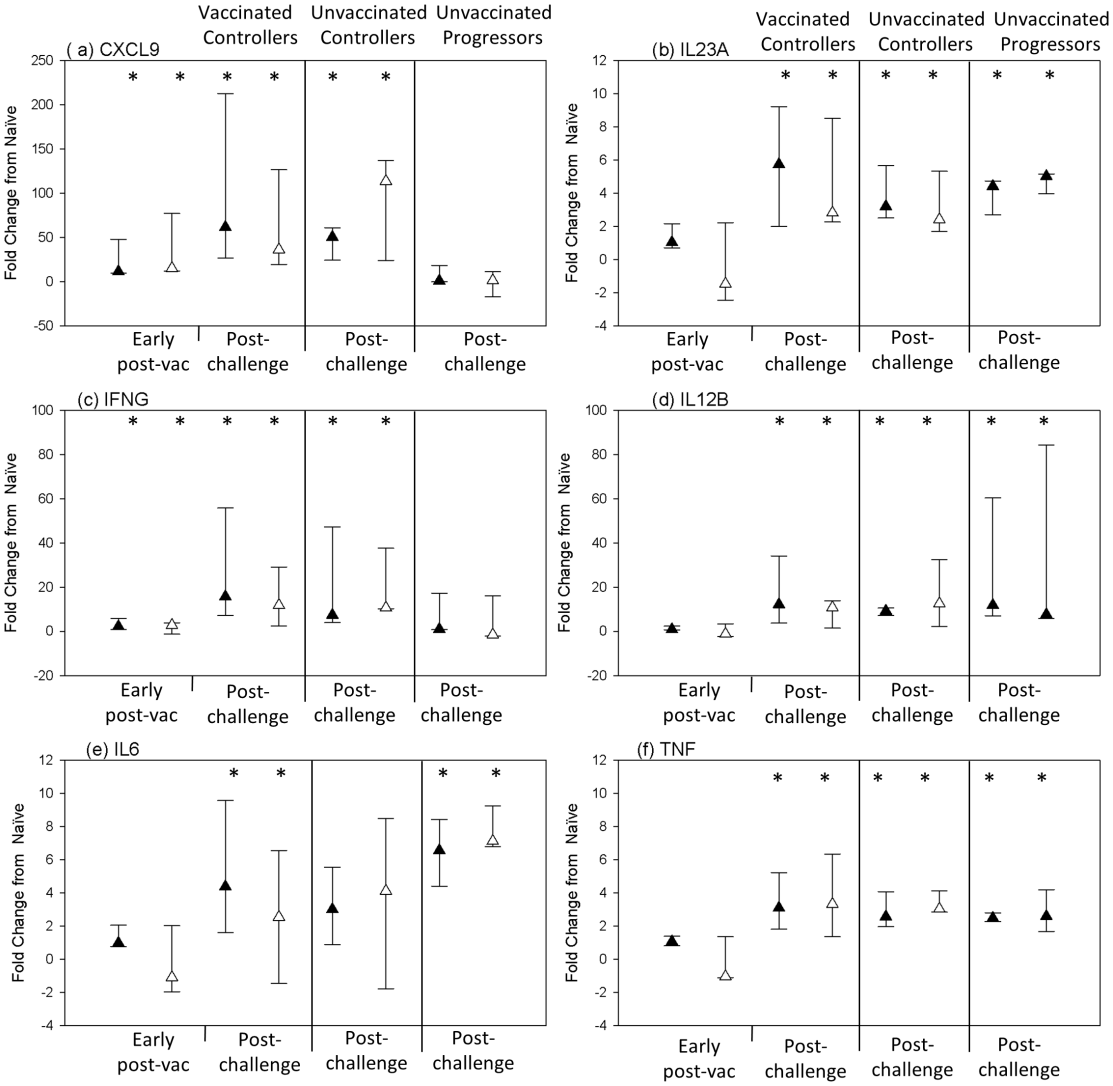
Comparison of gene expression profiles generated from microarray and RT-PCR analysis. RT-PCR was used to validate microarray expression data. The symbol represents the median and the error bars represent the range. Black triangles represent fold change in expression from naïve time-points as determined by microarray analysis; white triangles represent fold change in expression from naïve time-points as determined by RT-PCR analysis. * = differentially expressed (p<0.05).

To validate the microarray data, RT-PCR was used to determine the fold change in expression of six genes post-vaccination and/or post-challenge for all experimental groups, compared to expression at the naïve time-point. Student T tests were carried out on log_2_-transformed RT-PCR expression data to determine whether the genes were differentially expressed from the naïve time-point, with a p value <0.05 considered statistically significant. Expression profiles generated from the two techniques are shown in Figure 4. Time-points at which the genes were determined as differentially expressed are indicated on the plots. There was agreement for 100% of comparisons providing confidence in the microarray data obtained.

## Discussion

BCG is the only licensed vaccine for tuberculosis but offers variable protection, particularly against pulmonary tuberculosis, which is the most common form of the infection in adults; therefore, the development of a more effective vaccine is critical for the control of the on-going TB pandemic [21]. The identification of a definitive correlate of protection for TB would accelerate the process of clinical trials and pre-clinical assessments by acting as a surrogate end-point. Similarly, identification of biomarkers of TB disease could speed up the assessment of new anti-tuberculosis drugs and therapies [22].

Vaccine efficacy in TB NHP models, as with other *in vivo* models, is typically measured either as an increase in survival period following challenge, or as a reduction in TB-induced disease burden measured by changes in clinical parameters (such as weight loss, temperature increase, increase in markers of inflammation e.g. erythrocyte sedimentation rate (ESR), and decrease in red cell haemoglobin); organ-specific bacterial burden; or the number and extent of lesions in pulmonary and extra-pulmonary tissues measured using a qualitative gross pathology scoring system. The study report here was designed to use the comparative measures of disease burden (described above) applied 28 weeks after challenge, to evaluate the effect of BCG vaccination. The disease burden measured in the BCG vaccinated animals was significantly lower than that measured in the unvaccinated group (weight loss p = 0.0163; RBC haemoglobin p = 0.0051; ESR p = 0.0135; Total pathology score p = 0.0131) and therefore supports the conclusion that BCG vaccination leads to an improved outcome. The difference in rate of TB disease development between the BCG vaccinated and unvaccinated groups is reflected by ‘survival’, and approaches significance (Log rank analysis p = 0.055) which is compelling given the small numbers in the study and because the differences in rate of disease progression between groups, and individuals within groups, are in line with the differences revealed by the other measures of disease burden. The study provides a rare opportunity to relate differential gene expression patterns to different clinical outcomes to identify profiles associated with improved outcome of infection that are either naturally occurring (i.e. in unvaccinated animals) or induced by BCG vaccination.

The microarray approach allowed simultaneous analysis of the expression of tens of thousands of genes providing insights into all aspects of the host response to BCG-vaccination and TB-challenge. After BCG-vaccination, *IFNG* and *IL2* genes were up-regulated (Table 1), two cytokines which are vital for TB infection control. Also up-regulated was the proinflammatory cytokine *IL17,* which is produced by several cell types but is the primary cytokine secreted by a subset of CD4+ T cells (Th17 cells), which are important for vaccine-induced protection [23], [24]. IL17-producing T cells were generated in mice upon vaccination with a defined subunit TB vaccine [11]. These cells preferentially trafficked through or resided within the lungs, whereas a population of IFNG-producing T cells that was also generated typically resided within the central lymphoid tissue [11]. The position of these IL17-producing memory T cells within the lungs meant they were able to respond quickly when stimulated by antigen and produce IL17, leading to the recruitment of antigen-specific IFNG-producing T cells from the central lymphoid tissue back to the site of infection. This resulted in earlier cessation of bacterial growth and a reduced bacterial burden. The up-regulation of *IL17* expression in the BCG-vaccinated cynomolgus macaques, which controlled TB infection, supports a protective role for these cells. Furthermore, the expression of IL17 post-vaccination has been correlated with protection in a bovine model, in this case after BCG and a viral vectored booster vaccine [24].

In addition to the up-regulation of *IL17* post-vaccination in the vaccinated controllers, several genes associated with the Th17 immune response were also up-regulated at six weeks post-challenge in this group and also in the unvaccinated disease controllers. This observation was not evident in cynomolgus macaques that developed TB disease, further suggesting a potential protective role for these genes. In particular, the genes for *IL17, IL21, IL22, IL23A, IL26, CCL20* and *TNF*, which are either produced by, or required for the differentiation or maintenance of Th17 cells, were all up-regulated in cynomolgus vaccinated disease controllers. In addition, the expression of the lineage-specific transcription factors, *RORC* and *RORA*, were also significantly up-regulated in the cynomolgus vaccinated controllers. All these genes, with the exception of *IL26* and *RORC*, were also differentially expressed in the unvaccinated disease controllers but only *IL23Α, CCL20* and *TNF* were up-regulated in the cynomolgus disease progressors. Others have reported that Th17 cells and IL17 play an important role in the protective immune response to TB [25]–[27] and also that suppression of the Th17 response may occur in patients with active TB [28], [29]. Our microarray analysis revealed no differential expression of Th17-associated genes, including *IL17* or *IL22*, in the PBMCs of the cynomolgus macaques that developed active TB disease. This observation may be explained by the movement of cells that produce these cytokines out of the periphery and to the site of infection where, in these animals, *M. tuberculosis* has been able to establish an infection and, consequently, were able to suppress the protective Th1 and Th17 responses. One possible cause of this suppression could be an increase in levels of prostaglandin E_2_ in the animals in which TB disease progressed, as indicated by greater up-regulation of expression of genes involved in the synthesis of PGE_2_, such as *PTGS1*, *PTGS2* and *PTGES*, compared to that seen in disease controllers. While at low concentrations PGE_2_ can actually promote Th1 and Th17 cell differentiation [30]–[32] and enhance IL17 production [33], at greater concentrations, PGE_2_ has been shown to inhibit Th1 differentiation and T cell responses [34], [35].

The up-regulation of IL17 at 6 weeks post-challenge was observed in both vaccinated and unvaccinated disease controllers with no significant difference in expression between the two groups. This suggests that BCG-vaccination induces IL17 and a protective immune response that can control subsequent infection, as described above, but also some unvaccinated animals can produce IL17 by the natural route of innate, then adaptive immunity. As only one time-point was analysed post-challenge in this study, the time it takes to raise this response was not established. Therefore, it could be possible that vaccination results in a stronger, faster induction of the response with an instant boost to IL17 production, than that seen in the unvaccinated animals able to control infection. This would need to be investigated further.

A number of iron regulatory genes were differentially expressed only in the disease progressors, including genes expressing the heavy chain of ferritin, the divalent cation transporter, SLC11A1, and the transferrin receptor, which is important in moving iron bound to the extracellular iron storage protein, transferrin (TF), into the cell. Iron is required by the host to raise an adequate immune response as it is a cofactor for a number of metabolic enzymes and in the generation of reactive oxygen species. However, *M. tuberculosis* requires iron for its own metabolic processes and successful growth. Upon infection, there is competition between the host and bacteria over the iron in the body. When a pathogen is encountered, a non-specific acute phase response is induced within the host and iron is sequestered from invading pathogens by reducing absorption of iron by the gut (controlled by the protein, hepcidin) and by binding extracellular iron to storage molecules, such as transferrin, lactoferrin and ferritin. Mycobacteria deploy iron acquisition mechanisms to combat this and produce two types of high affinity iron-chelating siderophores; these are the cell-associated water-insoluble mycobactin and water-soluble carboxymycobactin, which is secreted and able to remove iron bound to host proteins and transport it back to the mycobacteria. In addition, a haem-degrading protein has recently been identified in mycobacteria, suggesting these bacteria can utilise free haem and haem from haemoglobin as an iron source [36].

Three of the six unvaccinated cynomolgus macaques were followed for a total of 28 weeks after *M. tuberculosis* challenge, having controlled infection for the duration of the study. The remaining three unvaccinated cynomolgus macaques reached humane end-point criteria before the end of the study and were euthanized. Their iron status was determined at post-mortem via expression analysis of a range of iron regulatory genes. A clear difference in expression of iron regulatory genes was observed with a consistent and significant down-regulation of *FTH1, SLC11A1, SLC11A2, HMOX1* and *TFRC* in animals controlling the infection (Figure 3). By reducing the storage protein ferritin, from which mycobacteria can obtain iron via the action of their siderophores, and reduction of the transferrin receptor, which would prevent iron bound to transferrin from entering the cell, the host actively restricts access of iron to the mycobacteria. At six weeks post-challenge, IFNG was up-regulated in animals that controlled infection. IFNG suppresses the expression of the transferrin receptor on monocytes and also down-regulates the intracellular concentration of ferritin [37], [38]. The down-regulation of both *FTH1* and *TFRC* in these animals could, therefore, be a result of the earlier actions of IFNG.

A role for iron in the control of TB disease has also been observed in guinea pigs [39]–[41]; the *ex vivo* infection of splenocytes isolated from protected BCG-vaccinated guinea pigs resulted in down-regulation of iron regulatory genes including the ferritin heavy chain and lactoferrin, and a heightened cytokine/IFNG response, which is similar to the trend reported here.

In animals that were unable to control infection, an up-regulation of IFNG was not observed at six weeks post-challenge; consequently, the high iron-transferrin in the blood caused by the acute phase response would result in the unidirectional and coordinated up-regulation of TFRC and intracellular ferritin content, and this was observed in samples taken at post-mortem during end-stage disease. SLC11A1 was also up-regulated in the unvaccinated disease progressors, as was SLC11A2, which localizes with transferrin in the recycling endosomes of a number of cell types and transports iron from the acidified lumen of the endosomes into the cytoplasm [42]. The increase in expression of genes involved in iron regulation seen in cynomolgus unvaccinated disease progressors had the overall effect of increasing the intracellular iron content which is available to the mycobacteria, allowing it to thrive and establish a successful infection.

As discussed above, the Th1 and Th17 responses found to be protective in the cynomolgus macaque model of TB in this study have also been shown to be protective against TB in other studies involving animals and also in humans [23]–[29]. However, the neutrophil-driven interferon response identified in human TB disease observed in the often cited study by Berry et al (2011) was not observed. [43]. This is most likely due to the fact whole blood was used to identify TB biomarkers in the Berry study whereas PBMCs, a fraction of blood depleted of neutrophils, was utilised here [43]. Nevertheless, despite this and other fundamental differences in experimental set up, it has been reassuring to see some genes appear across gene lists, highlighting the importance of these genes in the response to tuberculosis infection. Comparison of the 393-gene profile with differentially expressed genes in the cynomolgus macaques that developed and succumbed to TB disease revealed 61 genes in common, which is quite significant in view of the considerable differences in design between the two studies. Common genes included the immune-related genes CD274, IL1RN, Fc fragment of IgA, receptor for (FCAR), LILRA5, the interferon-inducible genes, CXCL10, GBP1 and IFI44L and the toll-like receptors, TLR2 and TLR5 [43].

## Conclusion

In conclusion, induction of a balanced Th1/Th17 response and production of key effector cytokines, such as IFNG, IL2, IL17, IL21, IL22 and IL23A was observed in animals that controlled infection, regardless of previous BCG-vaccination. The induction of a protective Th1 and Th17 response is influenced by many factors including the pro-inflammatory and inhibitory actions of cytokines and metabolites. Changes that affect the delicate balance between these factors could potentially sway the response in favour of a protective Th1/Th17 response or alternatively toward inhibition of these responses and, consequently, failure to control infection. The ability to adequately restrict access to iron from the mycobacteria is also an essential innate defence. The clear differences between disease controllers and progressors suggest that these responses could be used as indicators of protection or disease.

## Supporting Information

Table S1 **List of genes found to be differentially expressed in PPD-stimulated PBMCs isolated 6 weeks post-** ***M. tuberculosis*** **challenge in BCG-vaccinated cynomolgus macaques able to control infection (vaccinated controllers (n = 6)).**
(XLSX)

Table S2 **List of genes found to be differentially expressed in PPD-stimulated PBMCs isolated 6 weeks post-** ***M. tuberculosis*** **challenge in unvaccinated cynomolgus macaques able to control infection (unvaccinated controllers (n = 3)).**
(XLSX)

Table S3 **List of genes found to be differentially expressed in PPD-stimulated PBMCs isolated 6 weeks post-** ***M. tuberculosis*** **challenge in unvaccinated cynomolgus macaques unable to control infection (unvaccinated progressors (n = 3)).**
(XLSX)

## References

[1] WHO (2011) Global tuberculosis control: WHO report 2011. World Health Organization.

[2] Flynn J, Chan J, Triebold K, Dalton D, Stewart T, et al (1993) An essential role for interferon gamma in resistance to *Mycobacterium tuberculosis* infection. J Exp Med 178: 2249–2254. 7504064 10.1084/jem.178.6.2249 PMC2191274

[3] Gómez-Reino JJ, Carmona L, Valverde VR, Mola EM, Montero MD (2003) Treatment of rheumatoid arthritis with tumor necrosis factor inhibitors may predispose to significant increase in tuberculosis risk: A multicenter active-surveillance report. Arthritis Rheum 48: 2122–2127. 12905464 10.1002/art.11137

[4] Xing Z, Zganiacz A, Santosuosso M (2000) Role of IL-12 in macrophage activation during intracellular infection: IL-12 and mycobacteria synergistically release TNF-α and nitric oxide from macrophages via IFN-γ induction. J Leukoc Biol 68: 897–902. 11129658

[5] Ellner JJ, Hirsch CS, Whalen CC (2000) Correlates of protective immunity to *Mycobacterium tuberculosis* in humans. Clin Infect Dis 30: S279–S282. 10875800 10.1086/313874 PMC4515748

[6] Jeevan A, Bonilla DL, McMurray DN (2009) Expression of interferon-γ and tumour necrosis factor-α messenger RNA does not correlate with protection in guinea pigs challenged with virulent *Mycobacterium tuberculosis* by the respiratory route. Immunology 128: e296–e305. 19016908 10.1111/j.1365-2567.2008.02962.x PMC2753903

[7] Kagina BMN, Abel B, Scriba TJ, Hughes EJ, Keyser A, et al (2010) Specific T cell frequency and cytokine expression profile do not correlate with protection against tuberculosis after bacillus Calmette-Guérin vaccination of newborns. Am J Respir Crit Care Med 182: 1073–1079. 20558627 10.1164/rccm.201003-0334OC PMC2970848

[8] Sharpe SA, McShane H, Dennis MJ, Basaraba RJ, Gleeson F, et al (2010) Establishment of an aerosol challenge model of tuberculosis in rhesus macaques, and an evaluation of endpoints for vaccine testing. Clin Vaccine Immunol 17: 1170–1182. 20534795 10.1128/CVI.00079-10 PMC2916246

[9] Fletcher HA (2007) Correlates of immune protection from tuberculosis. Curr Mol Med 7: 319–325. 17504116 10.2174/156652407780598520

[10] Bold TD, Ernst JD (2012) CD4+ T cell-dependent IFN-γ production by CD8+ effector T cells in *Mycobacterium tuberculosis* infection. J Immunol 189: 2530–2536. 22837486 10.4049/jimmunol.1200994 PMC3424308

[11] Khader SA, Bell GK, Pearl JE, Fountain JJ, Rangel-Moreno J, et al (2007) IL-23 and IL-17 in the establishment of protective pulmonary CD4+ T cell responses after vaccination and during *Mycobacterium tuberculosis* challenge. Nat Immunol 8: 369–377. 17351619 10.1038/ni1449

[12] Meraviglia S, El Daker S, Dieli F, Martini F, Martino A (2011) γδ T cells cross-link innate and adaptive immunity in *Mycobacterium tuberculosis* infection. Clin Dev Immunol 2011. 10.1155/2011/587315 PMC3022180 21253470

[13] Sada-Ovalle I, Chiba A, Gonzales A, Brenner MB, Behar SM (2008) Innate invariant NKT cells recognize *Mycobacterium tuberculosis*–infected macrophages, produce interferon-γ, and kill intracellular bacteria. PLoS Path 4: e1000239. 10.1371/journal.ppat.1000239 PMC2588496 19079582

[14] Gold MC, Cerri S, Smyk-Pearson S, Cansler ME, Vogt TM, et al (2010) Human mucosal associated invariant T cells detect bacterially infected cells. PLoS Biol 8: e1000407. 20613858 10.1371/journal.pbio.1000407 PMC2893946

[15] Aranday Cortes E, Kaveh D, Nunez-Garcia J, Hogarth PJ, Vordermeier HM (2010) *Mycobacterium bovis* BCG vaccination induces specific pulmonary transcriptome biosignatures in mice. PLoS ONE 5: e11319. 20596522 10.1371/journal.pone.0011319 PMC2893133

[16] Langermans JAM, Doherty TM, Vervenne RAW, Laan Tvd, Lyashchenko K, et al (2005) Protection of macaques against *Mycobacterium tuberculosis* infection by a subunit vaccine based on a fusion protein of antigen 85B and ESAT-6. Vaccine 23: 2740–2750. 15780721 10.1016/j.vaccine.2004.11.051

[17] Lin PL, Dietrich J, Tan E, Abalos RM, Burgos J, et al (2012) The multistage vaccine H56 boosts the effects of BCG to protect cynomolgus macaques against active tuberculosis and reactivation of latent *Mycobacterium tuberculosis* infection. J Clin Invest 122: 303–314. 22133873 10.1172/JCI46252 PMC3248283

[18] McMurray D (2000) A non-human primate model for preclinical testing of new tuberculosis vaccines. Clin Infect Dis 30: S210–S212. 10875785 10.1086/313885

[19] Verreck FAW, Vervenne RAW, Kondova I, van Kralingen KW, Remarque EJ, et al (2009) MVA85A boosting of BCG and an attenuated *phoP* deficient *M. tuberculosis* vaccine both show protective efficacy against tuberculosis in rhesus macaques. PLoS ONE 4: e5264. 19367339 10.1371/journal.pone.0005264 PMC2666807

[20] Sharpe SA, Eschelbach E, Basaraba RJ, Gleeson F, Hall GA, et al (2009) Determination of lesion volume by MRI and stereology in a macaque model of tuberculosis. Tuberculosis 89: 405–416. 19879805 10.1016/j.tube.2009.09.002

[21] Ottenhoff THM, Kaufmann SHE (2012) Vaccines against tuberculosis: Where are we and where do we need to go? PLoS Path 8: e1002607. 10.1371/journal.ppat.1002607 PMC3349743 22589713

[22] Wallis RS, Doherty TM, Onyebujoh P, Vahedi M, Laang H, et al (2009) Biomarkers for tuberculosis disease activity, cure, and relapse. Lancet Infect Dis 9: 162–172. 19246020 10.1016/S1473-3099(09)70042-8

[23] Gopal R, Rangel-Moreno J, Slight S, Lin Y, Nawar HF, et al. (2013) Interleukin-17-dependent CXCL13 mediates mucosal vaccine–induced immunity against tuberculosis. Mucosal Immunol doi:10.1038/mi.2012.135. 10.1038/mi.2012.135 PMC3732523 23299616

[24] Vordermeier HM, Villarreal-Ramos B, Cockle PJ, McAulay M, Rhodes SG, et al (2009) Viral booster vaccines improve mycobacterium bovis BCG-induced protection against bovine tuberculosis. Infect Immun 77: 3364–3373. 19487476 10.1128/IAI.00287-09 PMC2715681

[25] Okamoto Yoshida Y, Umemura M, Yahagi A, O ’Brien RL, Ikuta K, et al (2010) Essential role of IL-17A in the formation of a mycobacterial infection-induced granuloma in the lung. J Immunol 184: 4414–4422. 20212094 10.4049/jimmunol.0903332

[26] Wozniak TM, Saunders BM, Ryan AA, Britton WJ (2010) *Mycobacterium bovis* BCG-specific Th17 cells confer partial protection against *Mycobacterium tuberculosis* infection in the absence of gamma interferon. Infect Immun 78: 4187–4194. 20679438 10.1128/IAI.01392-09 PMC2950338

[27] Desel C, Dorhoi A, Bandermann S, Grode L, Eisele B, et al (2011) Recombinant BCG ΔureC hly+ induces superior protection over parental BCG by stimulating a balanced combination of Type 1 and Type 17 cytokine responses. J Infect Dis 204: 1573–1584. 21933877 10.1093/infdis/jir592 PMC3192191

[28] Chen X, Zhang M, Liao M, Graner MW, Wu C, et al (2010) Reduced Th17 response in patients with tuberculosis correlates with IL-6R expression on CD4+ T cells. Am J Respir Crit Care Med 181: 734–742. 20019339 10.1164/rccm.200909-1463OC

[29] Cowan J, Pandey S, Filion L, Angel J, Kumar A, et al (2012) Comparison of IFNγ-, IL17- and IL22-expressing CD4 T cells, IL22-expressing granulocytes and proinflammatory cytokines during latent and active tuberculosis infection. Clin Exp Immunol 167: 317–329. 22236009 10.1111/j.1365-2249.2011.04520.x PMC3278699

[30] Yao C, Sakata D, Esaki Y, Li Y, Matsuoka T, et al (2009) Prostaglandin E2-EP4 signaling promotes immune inflammation through TH1 cell differentiation and TH17 cell expansion. Nat Med 15: 633–640. 19465928 10.1038/nm.1968

[31] Boniface K, Bak-Jensen KS, Li Y, Blumenschein WM, McGeachy MJ, et al (2009) Prostaglandin E2 regulates Th17 cell differentiation and function through cyclic AMP and EP2/EP4 receptor signaling. J Exp Med 206: 535–548. 19273625 10.1084/jem.20082293 PMC2699124

[32] Khayrullina T, Yen J-H, Jing H, Ganea D (2008) *In vitro* differentiation of dendritic cells in the presence of prostaglandin E2 alters the IL-12/IL-23 balance and promotes differentiation of Th17 cells. J Immunol 181: 721–735. 18566439 10.4049/jimmunol.181.1.721 PMC2835359

[33] Chizzolini C, Chicheportiche R, Alvarez M, de Rham C, Roux-Lombard P, et al (2008) Prostaglandin E2 synergistically with interleukin-23 favors human Th17 expansion. Blood 112: 3696–3703. 18698005 10.1182/blood-2008-05-155408 PMC2572797

[34] Cahill J, Hopper KE (1984) Immunoregulation by macrophages III prostaglandin E suppresses lymphocyte activation but not macrophage effector function during Salmonella enteritidis infection. Int J Immunopharmacol 6: 9–17. 6327542 10.1016/0192-0561(84)90029-8

[35] Murray JL, Kollmorgen GM (1983) Inhibition of lymphocyte response by prostaglandin-producing suppressor cells in patients with melanoma. J Clin Immunol 3: 268–276. 6224806 10.1007/BF00915351

[36] Tullius MV, Harmston CA, Owens CP, Chim N, Morse RP, et al (2011) Discovery and characterization of a unique mycobacterial heme acquisition system. Proc Natl Acad Sci USA 108: 5051–5056. 21383189 10.1073/pnas.1009516108 PMC3064333

[37] Byrd T, Horwitz MA (1989) Interferon gamma-activated human monocytes downregulate transferrin receptors and inhibit the intracellular multiplication of *Legionella pneumophila* by limiting the availability of iron. J Clin Invest 83: 1457–1465. 2496141 10.1172/JCI114038 PMC303847

[38] Byrd T, Horwitz M (1993) Regulation of transferrin receptor expression and ferritin content in human mononuclear phagocytes. Coordinate upregulation by iron transferrin and downregulation by interferon gamma. J Clin Invest 91: 969–976. 8450071 10.1172/JCI116318 PMC288049

[39] Thom RE, Elmore MJ, Williams A, Andrews SC, Drobniewski F, et al (2012) The expression of ferritin, lactoferrin, transferrin receptor and solute carrier family 11A1 in the host response to BCG-vaccination and *Mycobacterium tuberculosis* challenge. Vaccine 30: 3159–3168. 22426328 10.1016/j.vaccine.2012.03.008

[40] Tree JA, Patel J, Thom RE, Elmore MJ, Schäfer H, et al (2010) Temporal changes in the gene signatures of BCG-vaccinated guinea pigs in response to different mycobacterial antigens. Vaccine 28: 7979–7986. 20920573 10.1016/j.vaccine.2010.09.061

[41] Tree JA, Elmore MJ, Javed S, Williams A, Marsh PD (2006) Development of a guinea pig immune response-related microarray and its use to define the host response following *Mycobacterium bovis* BCG vaccination. Infection and Immunity 74: 1436–1441. 16428800 10.1128/IAI.74.2.1436-1441.2006 PMC1360318

[42] Gruenheid S, Canonne-Hergaux Fo, Gauthier S, Hackam DJ, Grinstein S, et al (1999) The iron transport protein NRAMP2 is an integral membrane glycoprotein that colocalizes with transferrin in recycling endosomes. J Exp Med 189: 831–841. 10049947 10.1084/jem.189.5.831 PMC2192949

[43] Berry MPR, Graham CM, McNab FW, Xu Z, Bloch SAA, et al (2011) An interferon-inducible neutrophil-driven blood transcriptional signature in human tuberculosis. Nature 466: 973–977. 10.1038/nature09247 PMC3492754 20725040

